# Materialism, affective states, and life satisfaction: case of Croatia

**DOI:** 10.1186/s40064-015-1494-5

**Published:** 2015-11-14

**Authors:** Ljiljana Kaliterna Lipovčan, Zvjezdana Prizmić-Larsen, Tihana Brkljačić

**Affiliations:** Ivo Pilar Institute of Social Sciences, Marulićev trg 19, 10000 Zagreb, Croatia; Washington University in St. Louis, St. Louis, USA

**Keywords:** Materialism, Life satisfaction, Positive affect, Negative affect

## Abstract

In recent years, a number of studies have used Material Values Scale (MVS) to assess beliefs about importance to own material things. The aims of this study were to validate the MVS scale and to explore the relationships between materialistic values and well-being of Croatian citizens. The study was carried out on a representative sample of N = 1129 Croatian citizens. We used the short 9-item version of the MVS, life satisfaction rating, ratings of two positive (Positive affect) and four negative emotions (Negative affect) over the past month, and demographic variables (age, gender, income). The original dimensionality of the MVS was not confirmed; confirmatory factor analyses yielded two instead of three factors, Happiness and Centrality/Success. When controlled for income, gender and age, the Happiness dimension predicted Life satisfaction and both Positive and Negative affect, indicating that people who believed that the material goods in ones life leads to happiness reported to have lower life satisfaction, lower level of positive affect and higher level of negative affect over the past month. The Centrality/Success dimension was positively related to Positive affect, indicating that the belief that possessions play a central role in enjoyment leads to more frequent experiences of happiness and satisfaction over the past month.

## Background

Materialism is generally considered a negative value, trait or behavior, being associated with greed, shallowness and lack of spiritual values. Collins English Dictionary defines materialism as “interest in and desire for money, possessions, etc., rather than spiritual or ethical values” (“Materialism” 2015). Studied within various disciplines materialism is defined from different perspectives: as a way of life, a value orientation, a cultural system, a personality trait, a second order value, an aspiration (Bindah and Othman 2011). There are two main approaches to materialism in the contemporary empirical research. One that views materialism as the personality trait and other that assumes that materialism should be treated as a part of personal value system.

Belk (1985) relates materialism to the personality traits of “possessiveness *(the inclination and tendency to retain control or ownership of one’s possessions)*, non-generosity *(an unwillingness to give possessions to or share possessions with others)* and envy *(displeasure and ill will at the superiority of another person in happiness, success, reputation, or the possession of anything desirable)”*. Fourth trait, preservation *(a tendency to make experience tangible through souvenirs and photographs)*, was added later, due to cross-cultural findings on materialism (Ger and Belk 1996). Belk (1984a, pp 291) defines materialism as “the importance a person attaches to material possessions and the belief that certain possessions are the primary source of happiness”. At the highest levels of materialism, possessions take a central place in person’s life and are believed to provide the greatest sources of satisfaction (Belk 1984b).

Another widely accepted approach to materialism suggest that materialism can be viewed as a value that consumers gives to possessions and should be studied within the context of the larger value systems that individuals hold (Richins and Dawson 1990; Kasser and Ryan 1996; Burroughs and Rindfleisch 2002). Richins and Dawson (1992, pp 308) define materialism as a “value that emphasis importance of possessions and material goods in person’s life toward achieving life goals or desired states”.

Implementing the basic concept of materialism as multi-faceted construct which includes the beliefs that possessions lead to happiness, that success can be judged by things people own, and that possessions are central in person’s life, Richins and Dawson (1992) constructed the Material Values Scale (MVS). The measure captures three dimensions of materialism: Success *(“I like to own things that impress people.”)*, Centrality *(“I enjoy spending money on things that aren’t practical.”)*, and Happiness *(“My life would be better if I owned certain things I don’t have.”)*. A review of published studies and analysis of fifteen data sets revealed that the MVS performs well in terms of reliability and empirical usefulness, but the dimensional structure proposed by authors is not always evident in the data (Richins 2004). In line with that, authors emphasized the need for cross-national validation of MVS as it was shown not to perform equally across different national samples. Specifically, it was found to have different factor structure in the French and Russian samples (Griffin et al. 2004) as well as in the German one (Müller et al. 2013).

### Materialism and well being

Materialism has drawn attention of scholars in the field of positive psychology because of its possible negative effects on individual’s well-being. In many studies conducted in different countries materialism was found to be associated with dissatisfaction with life and lower subjective well-being (Belk 1984b, 1985; Dawson and Bamossy 1991; Richins and Dawson 1992; Wright and Larsen 1993; Keng et al. 2000; Roberts et al. 2005; Ditmar et al. 2014). Moreover, materialism was found to be positively related to psychological illnesses such are paranoia and depression (Kasser and Ryan 1993). Regarding personal characteristics, materialistic persons were found to be socially anxious, self-conscious, and conforming (Schroeder and Dugal 1995), often concerned about appearances, and motivated by the extrinsic goals (Kasser and Ahuvia 2002). Materialism was also found to be related to anti-social behaviours such as conflicts between spouses (Paduska 1992) and tendency to engage in shoplifting (Larsen et al. 1999).

There are several interpretations of such findings. Some authors claim that materialists use material goods as compensation for some personal weaknesses like a low self-esteem (Chatterjee and Farkas 1992) or need for security and connectedness to others (Kasser et al. 1995). Other authors suggest that materialists set unrealistically high goals, so that the discrepancy between these expectations and actual achievement makes them unhappy (Sirgy 1998; Oishi et al. 1999). Such interpretation is in line with findings that even after substantial improvement of personal wealth, materialistic persons did not show an increase in subjective well-being (Ryan et al. 1999; Sheldon and Kasser 1998). As defined by Kasser and Ryan (1996) materialistic values include desire to be wealthy, to have possessions, to build a certain self-image, but also to be attractive, popular and well known. Such desires are not easy to achieve and materialistic people need to invest time and effort to accomplish their goals. In a recent study Kasser et al. (2014) experimentally manipulated materialism orientations in order to change well-being and found out that well-being increased as people placed less importance on materialistic values and goals. Conversely, as people increased the relative importance of materialistic values, well-being was found to decrease over time (Kasser et al. 2014). In series of studies, Solberg et al. (2004) provided several explanations for negative relation between materialism and well-being. First, they found that the relation could be partially explained by a confounding factor: neuroticism. Also, they proved that materialists tend to consider themselves less happy because of their poor social relations, and their tendency to work toward materialistic yet less enjoyable and harder-to-achieve goals.

Mentioned studies dominantly used life satisfaction as a measure of well-being. However, subjective well-being is multifaceted construct that consists of both cognitive and affective aspects. While cognitive component of well-being is usually conceptualized as life satisfaction, affective component reflect frequency of pleasant and unpleasant emotions (Diener 2006). In the meta-analysis of the relationships between materialism and personal well-being Dittmar et al. (2014) reported that materialism is associated with variety of well-being constructs, including one’s cognitive appraisals of overall life satisfaction as well as emotional appraisals of happiness and experiences of positive and negative emotions. However, the strength of associations between materialism and well-being differed for different constructs of well-being, although majority of associations were negative. A few studies so far surveyed experienced emotions (i.e. positive and negative affects) and their association with level of materialism (Christopher and Schlenker 2004; Christopher et al. 2009; Hudders and Pandelaere 2012). Most of these researchers agree that materialists experience more negative affect. However, the relation between materialism and positive affect states is not so clear, and except Christopher and Schlenker (2004) who found a negative impact of materialism on positive affect, the majority of studies found weak or non-significant relationships between materialism and positive affect.

When examining the relationships between materialism and well-being, it should be kept in mind that different economic and cultural circumstances can influence the meaning of materialism and values toward possessions and consumption within a society. As argued by Delhey (2010, p 81) “happiness tends to be pretty materialist in poorer places and more post-materialist in richer ones”. Comparing the data of 48 countries, the author showed that income and possessions are more important pillars for personal quality of life in poorer than in richer countries.

### Why Croatia?

So far, materialism as a value orientation was not systematically researched in Croatia. In the past it has been a society influenced by traditional and collectivist values placing emphasis on the family and community, rather than the individual (Radin 2002; Jankovic and Dittmar 2006). Croatia’s recent history has been marked by three politically important facts: the fall of communism (the first free elections in 1990), the declaration of independence from Yugoslavia (1991), and the Homeland War (1991–1995). Thus, in the relatively short time span Croatia has undergone a ‘triple transition’ from a single party system to a pluralist democracy, from a planned to a free market economy and from war to peace. In the context of war, the early years of transition were marked by hyperinflation, rising unemployment, widespread grey and black economy, fall in economic output and an increase of income inequality. Transitional problems, coupled with war-related problems, caused the country to experience a slower democratization process than many other post-communist countries (Eurofound 2007). Although member of EU since 2013, Croatian society is still experiencing the economic hardship and at the same time is exposed to global consumerism. In line with shortly reviewed research on materialism and well-being, we found important to explore these relationships in Croatian society that is still experiencing the slow economic growth. To the best of our knowledge, this was the first attempt to examine materialism, as the value that emphasis importance of possessions, on the nationally representative sample and to examine its relationships with well-being of Croatian citizens. In addition we intended to explore these relationships by assessing various components of the subjective well-being (life satisfaction, positive and negative affects).

The first goal of the present study was to translate MVS and to analyze the structure of the scale on the nationally representative sample of Croatian citizens. The basic question was whether the factor structure obtained on Croatian data corresponds to the three-factor model of Happiness, Success, and Centrality. Based on the results of other studies (Griffin et al. 2004; Müller et al. 2013) we hypothesized that if there was discrepancy between original three-factor-model of MVS and obtained data, it would be found for factors of Centrality and Success, as those factors in other research showed the lack of replication.

The second goal was to explore the relationships between materialism and affective (i.e., Positive and Negative Affect) and cognitive components (i.e. Life satisfaction) of subjective well-being. Our hypothesis was that materialism would be negatively associated with life satisfaction and experiences of positive affect, and positively with experiences of negative affect.

## Methods

### Participants

Participants were a representative sample of 1129 Croatian citizens. The sample ranged in age from 18 to 95 years (M = 48.2; SD = 17.97). They were chosen as a multi-stage probability-based sample of Croatian population. Two-stage stratification was used, by region and the size of residence, and addresses were randomly selected at each sampling point.

The sample consisted of 628 females (56 %). With regard to the participants’ income status which was defined as monthly income per household member, 186 (17 %) had less than 137 (17 %) Euro, 361 (33 %) had 137–273 Euro, 369 (34 %) had 274–546 Euro, and 184 (16 %) more than 546 Euro.

### Instruments

#### Materialism

The 9-item short version form of Material Values Scale (MVS; Richins 2004) was used in the current study to assess participants’ level of materialism. The scale was in the first step translated into Croatian language. In the second step backward translation was verified for discrepancies against the original form by bilingual translator to ensure the semantic, idiomatic, and conceptual equivalences of the items.

Participants had to answer how much they agree or disagree with the statements on a five-point Likert scales which ranged from 1 as “strongly disagree” to 5 as “strongly agree”. Original scoring provides scores on three factors of materialism: Success (example item: “*The things I own say a lot how well I’m doing in life.”)*, Centrality (example item: “*I like a lot of luxury in my life.”*) and Happiness (example item:”*I’d be happier if I could afford to buy more things.”*). The scale included three items to tap each of three factors.

#### Positive and negative affect

To assess the affective component of subjective well-being, the ratings of positive and negative affects were obtained by asking subjects about their specific emotions over the past month. They reported how often they experienced two positive (i.e., happy and satisfied) and four negative emotions (i.e., sad, angry, depressive and stressed) over the past month using a seven-point scale with range from 1 as “almost never” to 7 as “almost always”. Positive affect was calculated as a mean of two positive emotions and Negative affect as a mean of four negative emotions.

#### Life satisfaction

The measure of life satisfaction, as the global cognitive judgments of satisfaction with one’s life was used to assess the cognitive component of subjective well-being (Diener 2006). The subjects were asked “*All things considered, how satisfied are you with your life as a whole nowadays?*”. They rated their satisfaction with the life using an 11-point scale where 0 means “extremely dissatisfied” and 10 means “extremely satisfied”. The one-item measure was acquired from the European Social Survey Well-being module (Huppert et al. 2009) which was originally adapted from Diener and authors’ Satisfaction with Life Scale (SWLS; Diener et al. 1985).

### Procedure

The study was conducted in November/December 2007 as a part of a public opinion survey carried out by in-person interviews in the respondents’ homes. Trained persons who attended training sessions on the use of the questionnaire and procedures did interviews. The Troldahl-Carter method of selecting adult respondents within households was used (Troldahl and Carter 1964). The respondents were told that responses were anonymous.

### Data analyses

To test the three-factor model of MVS scale proposed by Richins and Dawson (1992; Richins 2004), to explore its structure and to find the best solution model for the Croatian data we have used confirmatory factor analysis (CFA). The CFA is frequently used to cross validate the factor structure of a scale or measure.

For CFA in the current study multiple fit indices were used to assess model fit, i.e. χ^2^, the Comparative Fit Index (CFI), Root Mean Square of Approximation (RMSEA) and p of Close Fit (PClose), and their standard cutoff recommendations were employed (Hu and Bentler 1999). In general, a non-significant χ^2^ (p > 0.05) suggests good model fit, as it indicates that the model does not differ significantly from the observed data. The value greater than 0.95 for a CFI and a RMSEA value of 0.05 or less suggest good fit. PClose greater than 0.05 is recommended, as a PClose value less than 0.05 suggests that RMSEA is significantly greater than its suggested cutoff of 0.05.

To examine the internal consistency of the MVS scale and its factors Cronbach’s alphas were calculated. Hierarchical regression analyses were employed to evaluate the relationship between each of the subjective well-being measures (Positive affect, Negative affect, Life satisfaction) with materialism dimensions, while controlling for the impact of a set of demographic variables (gender, age, income). We controlled for the demographics variables gender, age and income, as other research showed they were associated with well-being variables and materialism (see for review Dittmar et al. 2014; Dittmar and Pepper 1994). This ensured that the observed effect of materialism on well-being variables was independent of the effects of controlled variables.

All analyses were performed using Statistical Package for the Social Sciences (SPSS) version 22 with AMOS package.

## Results

### Structure of Material Values Scale (MVS)

The scale items of the MVS scale and descriptive statistics are displayed in Table 1. Inspection of descriptive statistics implies relatively low level of materialistic values in Croatian population. Only 3 out of 9 items outscore the theoretical mean of 3 (theoretical range is 1-5). According to the original scorings of the MVS scale (Richins 2004), the items relating to Happiness dimension show the relatively highest scores, while those relating to Centrality the lowest.

**Table 1:** Descriptive statistics of Material Values Scale (MVS) for the Croatian sample

MVS items (H, S, C)^a^	N	Mean (SD)
1. My life would be better if I own certain things I don’t have. (H)	1089	3.4 (1.29)
2. The things I own say a lot about how well I’m doing. (S)	1086	3.4 (1.28)
3. I’d be happier if I could afford to buy more things. (H)	1106	3.3 (1.34)
4. It bothers me that I can’t afford to buy things I’d like. (H)	1099	3.0 (1.30)
5. Buying things gives me a lot of pleasure. (C)	1097	3.0 (1.34)
6. I admire people who own expensive homes, cars, clothes. (S)	1102	2.0 (1.19)
7. I like to own things that impress people. (S)	1100	1.9 (1.12)
8. I like a lot of luxury in my life. (C)	1103	1.9 (1.15)
9. I try to keep my life simple, as far as possessions are concerned. (C)^b^	1102	1.8 (0.94)

^a^Dimensions of MVS (*H* Happiness; *S* Success; *C* Centrality) according to Richins (2004)

^b^Item 9 was reversed scored prior to analyses, so all items were interpreted such that a higher score means more materialism

Confirmatory factor analysis (CFA) was used to test the three factor structure of MVS specified by Richins and Dawson (1992) and the standardized CFA loadings estimates are presented in Table 2. The overall χ^2^ resulting from this model was significant 315.6 (df = 24; p < 0.01), CFI was 0.88, RMSEA was 0.01 and PClose was 0.00. Observed indexes of fit failed to meet standards of a “good fit” which indicated that specified three-factor model was inconsistent with observed data. Also, the correlation estimate between the constructs of Centrality and Success was extremely high (Φ = 0.97) indicating a lack of discrimination between those two dimensions. A similar trend was observed by the other authors when testing the scale on different national samples, for example, Griffin et al. (2004) on Russian and French samples, as well as Müller et al. (2013) on German sample. Richins and Dawson (1992) also reported high Φ coefficients among dimensions (0.75).

**Table 2:** Results of confirmatory factor analysis testing the original three-factor model of MVS based on the Croatian sample

MVS items (H, S, C)^a^	H^a^	S^a^	C^a^
4. It bothers me that I can’t afford to buy things I’d like (H)	0.62		
1. My life would be better if I own certain things I don’t have (H)	0.70		
3. I’d happier if I could afford to buy more things (H)	0.78		
6. I admire people who own expensive homes, cars, clothes (S)		0.51	
2. The things I own say a lot about how well I’m doing (S)		0.27	
7. I like to own things that impress people (S)		0.75	
9. I try to keep my life simple, as far as possessions are concerned (C)			−0.35
5. Buying things gives me a lot of pleasure (C)			0.55
8. I like a lot of luxury in my life (C)			0.82

The results of CFA: Φ (H,S) = 0.57; Φ (H,C) = 0.41; Φ (S,C) = 0.97 χ^2^ = 315.6, df = 24; CFI = 0.88; RMSEA = 0.1, PClose = 0.000

^a^Dimensions of materialism (H-Happiness; S-Success; C-Centrality) according to Richins (2004)

Our hypothesis that three-factor model of MVS would fit the Croatian data was not confirmed so we further explored the structure of the MVS on the Croatian sample. Our prediction, that if there would be a discrepancy, it would be found for the factors of Centrality and Success, was confirmed.

To further explore the structure of MVS we defined a two-factor model and tested by means of CFA. The results are presented in Table 3. Observed indexes of fit (χ^2^ = 52.6, df = 20, p > 0.05; CFI = 0.99; RMSAE = 0.038, PClose = 0.000) met the standards of a “good fit”. The original Happiness related items clustered together (items 3, 1, and 4) and with one Success item (item 2) which was integrated in factor Happiness defined the first factor. The items in general reflected the importance of material goods in making ones’ life happy.

**Table 3:** *Results of confirmatory factor analysis for two*-*factor model based on the Croatian sample*

MVS items (H,S,C)^a^	Happiness	Centrality/success
3. I’d happier if I could afford to buy more things. (H^a^)	0.80	
1. My life would be better if I own certain things I don’t have. (H^a^)	0.68	
4. It bothers me that I can’t afford to buy things I’d like. (H^a^)	0.61	
2. The things I own say a lot about how well I’m doing. (S^a^)	0.45	
7. I like to own things that impress people. (S^a^)		0.81
8. I like a lot of luxury in my life. (C^a^)		0.78
5. Buying things gives me a lot of pleasure. (C^a^)		0.45
9. I try to keep my life simple, as far as possessions are concerned. (C^a^)		−0.40
6. I admire people who own expensive homes, cars, clothes. (S^a^)		0.33

The results of CFA: Φ (H,C/S) = .42; χ^2^ = 52.6, df = 20, p > .05; CFI = 0.99; RMSEA = 0.04, PClose = .000

^a^Dimensions of materialism (*H* Happiness; *S* Success; *C* Centrality) according to Richins (2004)

The second factor was best explained by two items (items 7 and 8) one of which was designed to capture Centrality, and the other Success. However, the remaining two Centrality items and one Success item (items 5, 9, and 6) loaded on both factors, although most of them scored higher on the second factor. Richins and Dawson (1992) reported also high Φ coefficient among those factors (up to 0.75). The meaning of those items in general reflected a tendency to spend and enjoy owning luxury things and that possession had a central role in ones’ life.

Therefore, we decided to score MVS for two factors that we defined as (1) *Happiness dimension* since it captured all original happiness items and one success item, and (2) *Centrality/Success dimension* as it captured all centrality items and two success items. Internal consistency analysis yielded Cronbach alpha for Happiness dimension 0.72, and Centrality/Success dimension 0.72.

### Relationship between materialism and subjective well-being

Mean scores and SD in subjective well-being measures and materialism dimensions based on two-factor model obtained on Croatian data are reported in Table 4.

**Table 4:** Descriptive statistics of subjective well-being and materialism dimensions in the Croatian sample

Measures	N	Mean (SD)
Life satisfaction	1129	6.8 (2.04)
Positive affect	1119	4.9 (1.10)
Negative affect	1121	3.1 (1.12)
Happiness (4 items)	1112	3.3 (0.96)
Centrality/Success (5 items)	1112	2.2 (0.80)

To explore the associations between well-being measures (Life satisfaction, Positive affect, Negative affect) and materialism (Happiness and Centrality/Success dimensions), hierarchical regression analyses were conducted separately on each of the well-being variables with age, gender and family income as covariates. The covariates were entered at first step while Happiness and Centrality/Success scores were entered in the second step of the analyses. Table 5 summarizes the results of the hierarchical regression analyses which contains standardized coefficients beta (ß), R square changes associated with each of the steps (R^2^ changes), adjusted R^2^ for each step, and multiple R of the final model for each well-being variables.

**Table 5:** Summary of hierarchical regression analyses for happiness and centrality/success predicting Life satisfaction, positive and negative AFFECT

	Life satisfaction β	Positive affect β	Negative affect β
Step 1			
Gender^a^	−0.02	−0.02	0.17**
Age	−0.15**	−0.21**	−0.10**
Income	0.25**	0.17**	−0.10**
R^2^ change	0.10**	0.09**	0.05**
Adjusted R^2^	0.10**	0.09**	0.05**
Step 2			
Gender^a^	−0.02	−0.02	0.17**
Age	−0.16**	−0.19**	−0.09**
Income	0.21**	0.14**	−0.06*
Happiness	−0.17**	−0.17**	0.20**
Centrality/success	0.01	0.09**	−0.01
R^2^ change	0.03**	0.02**	0.04**
Adjusted R^2^	0.12**	0.10**	0.08**
Multiple R	0.35**	0.33**	0.29**

^a^Gender 1 = Men, 2 = Women

** p < 0.01; * p < 0.05

The hierarchical regression analysis with Life satisfaction showed that multiple R for the final model was 0.35 (p < 0.01). Overall, all variables in the model explained 12 % of the variance in Life satisfaction, which was significant but low. Based on R square change, the Happiness and Centrality/Success dimensions predicted only 3 % of the variance in the Life satisfaction over and above the set of demographic variables (which alone accounted for 10 % of the variance in Life satisfaction). In other words, the contribution to the explanation of the variance in Life satisfaction was larger for set of demographic variables than for the MVS dimensions. After controlling for the relationship of demographic variables to the Life satisfaction, only Happiness dimension emerged as significant predictor of Life satisfaction (ß = −0.17). People who believed that consumption and material goods leads to happiness reported to have lower life satisfaction.

The hierarchical regression analysis with Positive affect showed that multiple R for the final model was 0.33 (p < 0.01). While all variables in the model explained 10 % of the variance in the Positive affect, again the contribution was larger for set of demographic variables (9 %) than for the MVS dimensions (2 %). After controlling for the demographic variables, the unique impact of two MVS dimensions on the Positive affect was observed. Happiness (ß = −0.17) and Centrality/Success dimensions (ß = 0.09) emerged as significant predictors of Positive affect. People who believed that material goods in ones’ life and being able to acquire them leads to happiness reported lower level of Positive affect, while those to whom possessions had a central role in their life reported higher levels of Positive affect.

The final hierarchical regression analysis with Negative affect showed that multiple R for the whole model was the lowest 0.29 (p < 0.01). While all variables in the model explained 8 % of the variance in the Negative affect, the contribution for set of demographic variables (5 %) and for the MVS dimensions (4 %) was quite similar. After controlling for demographic variables, only Happiness dimension (ß = 0.20) emerged as a significant predictor of Negative affect. People who believed that material goods and being able to acquire them leads to happiness reported higher levels of Negative affect.

Our second hypothesis was partly confirmed. Namely, Happiness dimension of materialism was negatively associated with life satisfaction and experience of positive affect, and positively associated with experience of negative affect. However, dimension of Centrality/Success was positively associated with positive affect only.

## Discussion

Much of the previous research on materialism used the Material Values Scale (Richins and Dawson 1992; Richins 2004), which was developed to capture the value that emphasize the importance of possessions and material goods in one’s life. The scale comprises three factors: Success, Centrality, and Happiness and has been used world-wide in the past three decades, mostly in marketing research (Sinkovics and Holzmuller 2001). There have been several studies aimed at validating and exploring the structure of MVS in different countries and cultural contexts (Griffin et al. 2004; Müller et al. 2013; Dittmar et al. 2014).

The current study was designed to validate the MVS, and to examine its psychometric properties in a Croatian sample, and to examine the relationships between dimensions of materialism and well-being in Croatian society. To the best of our knowledge, this is the first attempt to validate the scale in the Croatian population and to examine the importance of materialism, i.e. the value that emphasizes the importance of possessions, in a nationally representative sample of Croatian citizens.

Our first hypothesis was that the factor structure obtained in the Croatian data would correspond to the three-factor model of Happiness, Success, and Centrality, consistent with prior studies. Based on the previous research on dimensionality of MVS, we additionally hypothesized that, if there was discrepancy between original three-factor-model of MVS and obtained data, the discrepancy would be found for the factors of Centrality and Success. The confirmatory factor analysis conducted to test the Richins and Dawson’ model of MVS did not confirm the existence of three-factor structure in the Croatian sample. As we predicted the discrepancy was found for the factors of Centrality and Success as they displayed a lack of discrimination. Similar results were obtained by Griffin et al. (2004) when analysing a Russian sample of 103 participants using the 18-item MVS version, and by Müller et al. (2013) when analysing German sample of 2520 participants using 15-items MVS version. In all these analyses the Happiness dimension of MVS showed relatively stable structure across the different nationality samples, while Centrality and Success dimensions merged into one dimension.

The further analyses of the Croatian data proved that MVS was best represented by a two factors structure. The first factor included all original Happiness and one Success item, which in general reflected the importance of material goods in making ones’ life happy, so we view it as representing a Happiness dimension. All of the Centrality- and the rest of the Success- items loaded on the second factor, which generally reflected a tendency to spend money and enjoy owning luxury things. Thus we named it the Centrality/Success dimension. Both dimensions showed good internal consistency.

The Happiness dimension had a higher mean level than Centrality/Success in our sample (Table 4). This was also found in another Croatian sample in research that was aimed to compare materialistic values of students in Croatia, Germany and the UK (Jankovic and Dittmar 2006). Croatian students (N = 192) were found to score higher on Happiness than German and UK students. We argue that this dimension reflects not only a materialistic orientation, but is also connected to one’s level of wealth. For someone who owns nothing or very little it is logical that “his/her life would be better if owns certain things that doesn’t have”, would be “happier if could afford to buy more things” and “bothers him/her if cannot afford to buy things that likes”. These three items that define the Happiness dimension of the MVS are also descriptive of a poor person. When applied to someone who is well off, the same items describe someone who wants more things to be happy, i.e. someone with an obviously materialistic orientation. Therefore, we suggest that, when applied to individuals and/or societies with low income, this construct doesn’t exclusively reflect materialism, but also reflect a condition of poverty or low socioeconomic conditions. On the other side, the Centrality and Success items describe persons who like luxury, enjoy shopping (Centrality), or want to impress others (Success), and thus are not discriminative enough in a lower SES population to form two distinctive factors. Therefore, the specific economic context of Croatia, where a significant percent of population struggles to make ends meet, probably affected the model structure of the MVS in our sample. As argued by some authors, many items of MVS are based on assumptions that goods are easily obtained, which is not the case in all countries around the world (Griffin et al. 2004), and definitely not the case in Croatia.

Keeping in mind that materialism in lower SES population may reflect actual financial deficit in fulfilling basic needs, in further analyses we explored the relation between materialism and well-being. Our hypothesis was that materialism would be negatively associated with life satisfaction and positive affect experience, and positively with negative affect experience. Results showed that the Happiness dimension of MVS was consistently a significant predictor of all well-being measures. We found that people who hold the belief that possessions lead to happiness do experience lower levels of well-being in form of lower life satisfaction, higher negative affect and lower positive affect over the past month. These results are in accordance with previous research suggesting that materialism can lead to diminished well-being (e.g. Richins and Dawson 1992; Sirgy 1998; Christopher et al. 2009; Keng et al. 2000). In particular, materialists experience more negative feelings and lower life satisfaction compared to less materialistic persons (Deckop et al. 2010; Karabati and Cemalcilar 2010). Roberts and Clement (2007) found a negative relation between life satisfaction and all three dimensions of materialism, while in our sample only the Happiness dimension proved to be related to lower life satisfaction. However, it should be stressed that demographic variables explained much more variance in life satisfaction (10 %) than MVS scores (3 %). Among demographic variables, the most significant one was income (*β* = 0.24, p < 0.01), indicating higher life satisfaction in people with higher income. Similar result was also found in our previous research on Croatian citizens in 2005 (Kaliterna Lipovčan et al. 2007). Although income was controlled for when analysing relationships between dimensions of MVS and well-being measures, the fact that the Happiness dimension was found to be related to all three well-being measures again shows the possibility that this dimension reflects not only the materialistic value but economic deprivation, or low SES conditions as well, as discussed earlier.

Another result of this study was that the Centrality/Success dimension of MVS was related to positive affect, i.e. people who believed that possessions play a central role in personal gratification experienced higher frequency of positive emotions over the past month. A few studies that explored the relation between materialism and positive affect arrive at inconsistent results (Christopher and Schlenker 2004; Solberg et al. 2004; Hudders and Pandelaere 2012). For example, Solberg and colleagues (2004) found that pursuing financial goals directly relates to lower levels of positive affect. Hudders and Pandelaere (2012) found that materialists experience negative feelings more often than non-materialists, while a relation with positive feelings was not found. However, the same authors found that luxury consumption, i.e. actual behavior, was positively related to both, materialism and subjective well-being (positive feelings and general life satisfaction). Although in our research we did not examine luxury consumption, it is possible that the Centrality/Success dimension captures that behavior in a larger amount than Happiness. This could be one of the explanations of the positive relation between the Centrality/Success dimension and Positive affect. It is possible that enjoying luxury products gives materialists additional sources of positive affective states, which non-materialists do not experience. If such pleasures are momentary, then it is also possible that they do not influence other components of subjective well-being, especially the cognitive one such as life satisfaction.

## Conclusions

The validation of the MVS on a representative sample of Croatian citizens showed two- instead of three-factor solution. One dimension was labeled as Happiness as it captured beliefs that owing material goods and being able to acquire them leads to happiness, and another as Centrality/Success, capturing the believes that possessions are important for personal gratification. By the rated level of Happiness we suggest that perceptions of materialism in Croatian society may be different from the perception of materialism in societies that are more well off. The belief that owing material goods and being able to acquire them leads to happiness among Croatian citizens might not reflect materialistic values per se, but rather the expression of economic deprivation and inability to fulfill basic financial security. When examining the relationships between two MVS dimensions and well-being variables, it turned out that Happiness dimension was significantly related to life satisfaction, positive and negative affect, while Centrality/Success was significantly related only to positive affect. People who believed that owing material goods leads to happiness experienced lower levels of life satisfaction and positive affect, and higher levels of negative affect. People who believed that possessions are important for personal gratification experienced higher levels of positive affect. However, it should be noted that socio-demographic variables, especially income, that were controlled for when analyzing these relationships, played more important role in explaining well-being variables, than materialistic values.

This study is relevant as it is the first attempt to measure materialistic values on the large representative sample of Croatian citizens. In examining well-being we used both cognitive (life satisfaction) and affective components (experienced positive and negative emotions in the last month). However, our study also has some weaknesses. The present findings are based on cross-sectional and correlational data that limit inferences about processes and causality. Further research will be useful to refine the psychometric properties of the Material Values Scale. The factor structure of the scale that we obtained in our sample could be due to measurement artifacts, ambiguity, or true cultural differences in the measured construct, so distinguishing between these alternative explanations deserves further research. Also, studies on new data conducting CFA to confirm the obtained MVS structure on Croatian data are recommended. Further research should be aimed at comparing materialistic values across nations that have different socioeconomic and/or cultural characteristics, as socioeconomic conditions may moderate the relationships between materialism and other variables.
